# From ear to uncertainty: vestibular contributions to cognitive function

**DOI:** 10.3389/fnint.2013.00084

**Published:** 2013-11-26

**Authors:** Paul F. Smith, Yiwen Zheng

**Affiliations:** Department Pharmacology and Toxicology, School of Medical Sciences, and the Brain Health Research Centre, University of OtagoDunedin, New Zealand

**Keywords:** vestibular, spatial memory, cognition, hippocampus, vestibular lesions

## Abstract

In addition to the deficits in the vestibulo-ocular and vestibulo-spinal reflexes that occur following vestibular dysfunction, there is substantial evidence that vestibular loss also causes cognitive disorders, some of which may be due to the reflexive deficits and some of which are related to the role that ascending vestibular pathways to the limbic system and neocortex play in spatial orientation. In this review we summarize the evidence that vestibular loss causes cognitive disorders, especially spatial memory deficits, in animals and humans and critically evaluate the evidence that these deficits are not due to hearing loss, problems with motor control, oscillopsia or anxiety and depression. We review the evidence that vestibular lesions affect head direction and place cells as well as the emerging evidence that artificial activation of the vestibular system, using galvanic vestibular stimulation (GVS), can modulate cognitive function.

## Introduction

Recent epidemiological studies have demonstrated that vestibular disorders occur in more than 35% of adults aged 40 or older; between the ages of 60 and 69, the prevalence increases to almost 50% and between 70 and 79, it is 69% (Agrawal et al., 2009). Saber Tehrani et al. (2013) has estimated that, of 3.9 million patients visiting a Hospital Emergency Department for dizziness or vertigo in the USA in 2011, 25.7% were attributable to otological or vestibular causes, costing US $757 million. Vestibular dysfunction therefore represents a substantial and increasing burden on healthcare systems.

The most obvious effects of poor vestibular function are oscillopsia and ataxia (see Curthoys and Halmagyi, 1995 for a review); however, vestibular dysfunction involves a more complex syndrome characterized not only by reflex deficits, but also by attention and memory deficits, and anxiety disorders (see Smith et al., 2010 for reviews). It is evident that, in addition to the role of the vestibular system in the vestibulo-ocular and vestibulo-spinal reflexes (VORs and VSRs), the vestibular information provided in the ascending pathways to the limbic system and neocortex is required for an accurate internal representation of the relationship between the self and the spatial environment (Angelaki et al., 2009; Chen et al., 2013). In the absence of this information, this internal representation becomes inaccurate, ambiguous, and cognitive performance is affected. The aim of this review is to summarize and critically evaluate the current literature relating to the effects of vestibular function on cognition.

## Animal studies of the effects of vestibular lesions on memory

Many of the early animal studies of spatial navigation suggested that non-visual, idiothetic cues such as vestibular and proprioceptive information, along with external, allocentric cues, were used by animals in order to remember how to navigate their way through a familiar environment (Beritoff, 1965; Potegal et al., 1977; Etienne, 1980; Mittelstaedt and Mittelstaedt, 1980; Horn et al., 1981; Potegal, 1982; Miller et al., 1983; Etienne and Jeffery, 2004). It was speculated that vestibular information must be transmitted to the hippocampus, in order to be integrated with other sensory information relevant to spatial memory (Wiener and Berthoz, 1993; Berthoz, 1996; McNaughton et al., 1996; Etienne and Jeffery, 2004). Ultimately it was reported that place cells in the hippocampus, that respond to specific places in the environment, were modulated by vestibular stimulation (Gavrilov et al., 1995; Wiener et al., 1995), which was supported by numerous animal behavioral studies showing that the disruption of normal vestibular function resulted in spatial memory deficits (Potegal et al., 1977; Horn et al., 1981; Potegal, 1982; Miller et al., 1983; Petrosini, 1984; Mathews et al., 1988, 1989; Semenov and Bures, 1989; Chapuis et al., 1992; Ossenkopp and Hargreaves, 1993; Stackman and Herbert, 2002; Wallace et al., 2002; Russell et al., 2003a; Zheng et al., 2003, 2006, 2007, 2008, 2009a,b, 2012a,b; Baek et al., 2010; Besnard et al., 2012; Machado et al., 2012a,b; Smith et al., 2013).

**Figure 1:**
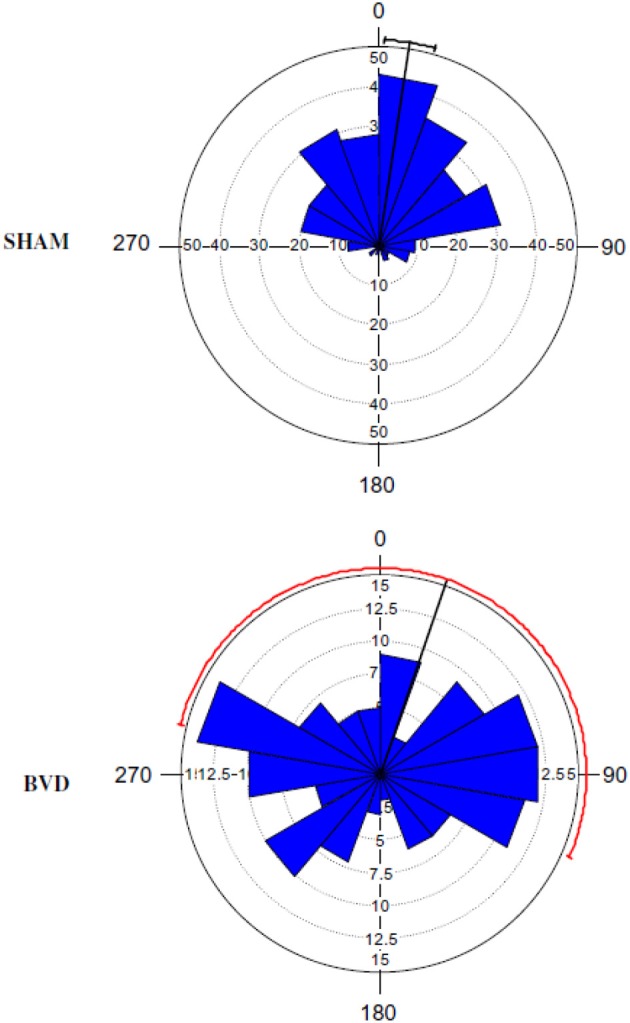
**A Rose diagram indicating the initial heading angles of SHAM and BVD rats at 14 months post-op. in a foraging task in darkness, in which they had to remember their way to a home base**. The mean vector is indicated by the black line and the 95% confidence interval (CI) for the mean is indicated by the line extending either side. The 95% CIs for the BVD animals were unreliable due to the low concentration of vectors. The inner circles (dotted line) indicate the number of observations for the given vectors (blue triangles). Reproduced with permission from Baek et al. (2010).

**Figure 2:**
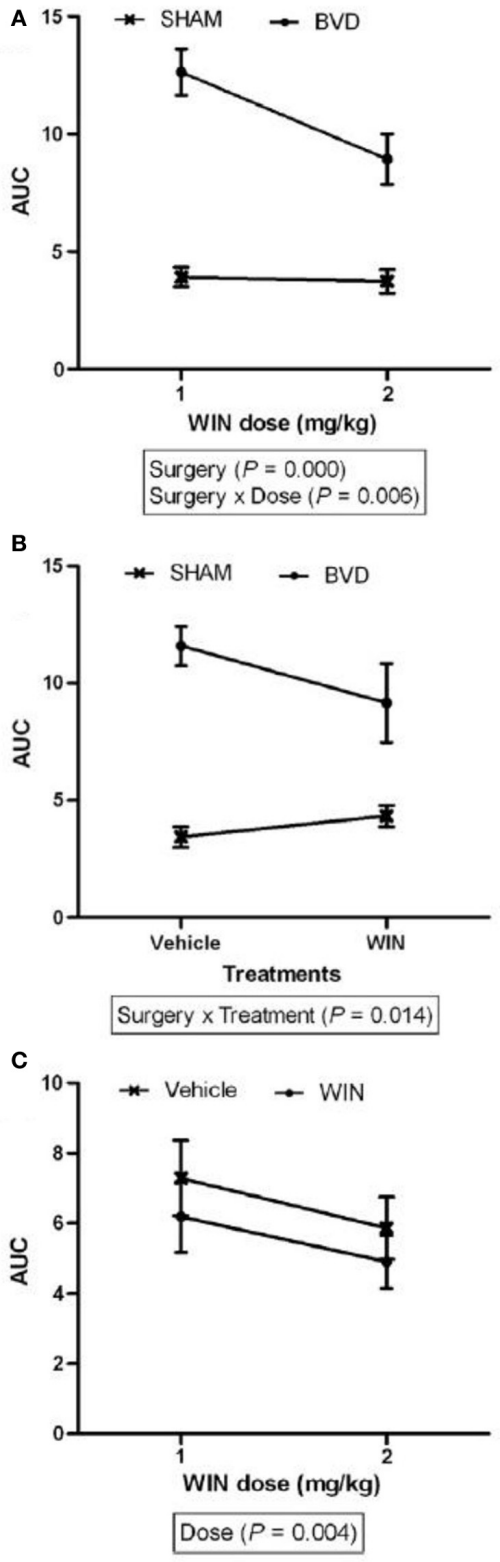
**The area under the curve (AUC) for the number of errors exhibited in the same foraging task described in Figure 1, for the sham and BVD animals at 14 month post-op. in darkness, showing the effects of surgery, drug treatment with a cannabinoid receptor agonist, WIN55,212-2 (which one would normally expect to make spatial memory worse), and their interaction. (A)** Effects of surgery; **(B)** Drug treatment; and **(C)** Drug dose. Data are represented as mean ± s.e.m. Reproduced with permission from Baek et al. (2010).

The early studies were open to the interpretation that what appeared to be spatial memory impairment following vestibular damage might be a direct result of oscillopsia, due to VOR deficits, or ataxia, due to VSR deficits. Such deficits never completely compensate even following a unilateral vestibular lesion (Smith and Curthoys, 1989; Curthoys and Halmagyi, 1995); therefore, this was a reasonable possibility. The first sophisticated study of spatial memory following bilateral vestibular lesions was reported by Wallace et al. (2002), who employed a foraging task, in which rats had to remember their way back to a home base, at 2 weeks post-op. This study, which used chemical lesions of the vestibular labyrinth with intratympanic sodium arsanilate, was well-controlled and used an electronic tracking system to quantify the rats' behavior. They found that rats with bilateral vestibular deaffferentation (BVD) exhibited profound spatial memory deficits in darkness, when visual cues were not available. Over the last decade in particular, many such studies have been conducted at much longer points after the lesion. Some compensation for the vestibular reflex deficits has occurred in the intervening period and yet the spatial memory deficits still persist (Zheng et al., 2006, 2007, 2008, 2009a,b, 2012a,b; Baek et al., 2010). In the study by Baek et al. (2010), which employed the longest post-operative time interval to date, rats that were 14 months post-BVD were more severely impaired in a spatial memory foraging task in darkness than at 5 months post-op. (see Figures 1, 2).

### Effects of UVD vs. BVD in a spatial memory task in darkness

Especially important is that the spatial memory deficits exhibited by BVD rats were substantially more severe than for animals with unilateral vestibular deafferentation (UVD) (Zheng et al., 2006, 2009b). Rats with UVD showed spatial memory deficits in darkness at 3 months following the lesion, but performed at levels similar to sham controls at 6 months post-op. (Zheng et al., 2006). However, at 5–6 months post-op., while BVD rats had only minimally impaired performance in a foraging task in light, their performance deteriorated substantially in darkness (Zheng et al., 2009b). By 14 months post-op., rats with the same kind of BVD lesions were severely impaired in darkness (Baek et al., 2010; see Figures 1, 2). In this latter study, even treatment with a cannabinoid receptor agonist, which would normally be expected to cause spatial memory impairment, could not increase the severity of the spatial memory deficits (Figure 2). These deficits in darkness suggest that oscillopsia is not necessary for the spatial navigation impairment to occur.

### Spatial memory deficits in light for BVD rats

**Figure 3:**
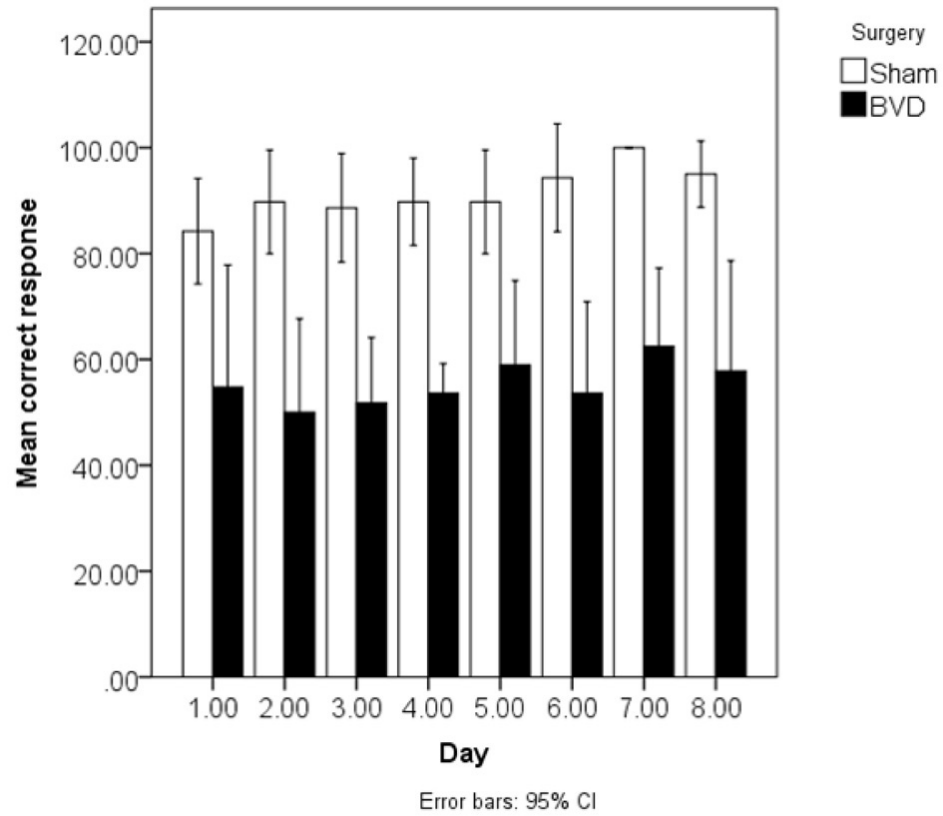
**Mean % correct responses in the spatial T maze task in light over 8 days for the BVD and sham animals at 4–5 months post-op**. Reproduced with permission from Zheng et al. (2012a,b). Data are expressed as means ± a 95% confidence interval.

BVD rats have also been demonstrated to exhibit spatial memory deficits in light. At 6 weeks post-op., rats with BVD performed significantly worse than sham controls in a radial arm maze task even in light (Russell et al., 2003a,b). During spatial alternation in a T maze task in light, rats with BVD exhibited some improvement in performance over time, but at 5 months post-op. their percentage of correct responses was still significantly below normal (Zheng et al., 2007). This result was recently replicated by Zheng et al. (2012a,b), using rats at 4–5 months following BVD (see Figure 3).

**Figure 4:**
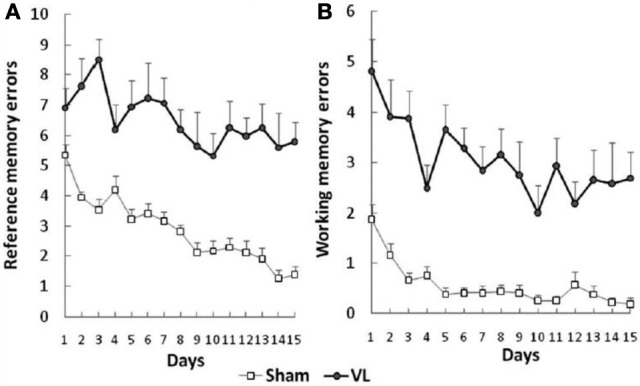
**Effects of sequential unilateral vestibular lesions on performance of rats, compared to sham controls, in a radial arm maze task. (A)** Number of reference memory errors. **(B)** Number of working memory errors. Data are represented as mean ± s.e.m. Reproduced with permission from Besnard et al. (2012).

**Figure 5:**
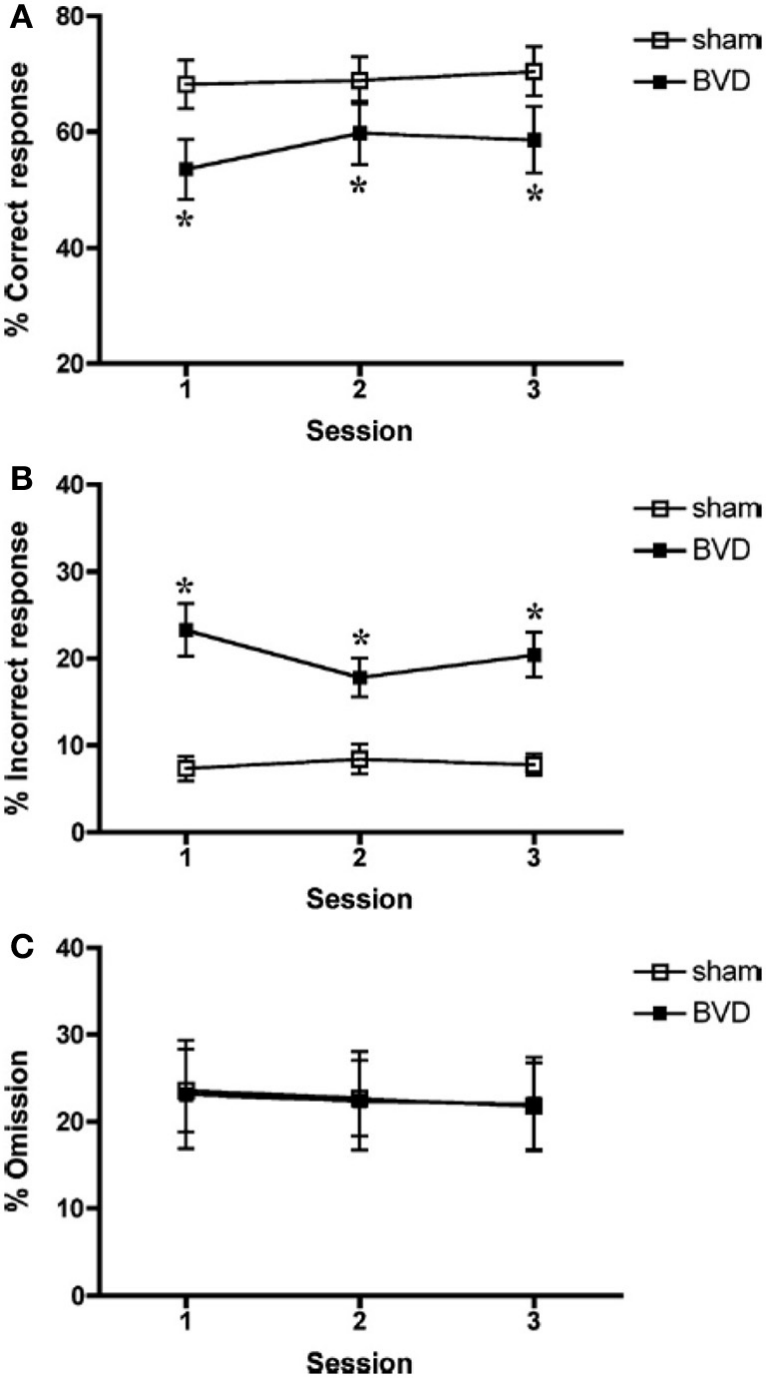
**Percentage of correct responses (A), incorrect responses (B), and omissions (C) for sham (open square) and BVD rats (closed square) in a 5 choice serial reaction time task in light**. Data are expressed as means ± s.e.m. Asterisks indicate significant differences. Reproduced with permission from Zheng et al. (2009a,b).

Besnard et al. (2012) and Machado et al. (2012a,b) have recently demonstrated similar spatial memory deficits in light using the radial arm maze and Y maze in rats that had sequential unilateral chemical labyrinthectomies using sodium arsanilate (Figure 4). In an automated 5 choice serial reaction time task (5-CSRTT), which is a task commonly used to assess attentional performance in light, rats with BVD made significantly fewer correct responses, significantly more incorrect responses, with no more omissions, and responded with a reduced latency, compared to sham controls (Zheng et al., 2009a,b; see Figure 5). This study in particular shows that the deficits of BVD rats in these cognitive tasks is not due to an inability to respond, but to incorrect responses.

These studies demonstrate that bilateral vestibular lesions have different effects in different cognitive tasks (i.e., radial arm maze, foraging task, Y and T mazes and 5-CSRTT) and that poor performance is not necessarily contingent upon the animal being in darkness, or light, where the oscillopsia would be expected to be worse, or due to an inability to respond. Therefore, the deficits are more likely to be due to an interaction between reduced cognitive ability and altered sensory input than altered sensory input alone. Studies over the last few years have aimed at elucidating the extent to which the spatial memory deficits may be due to other complications of BVD, such as changes in locomotor activity and anxiety.

### Possible relationship between spatial memory deficits and locomotor hyperactivity

**Figure 6:**
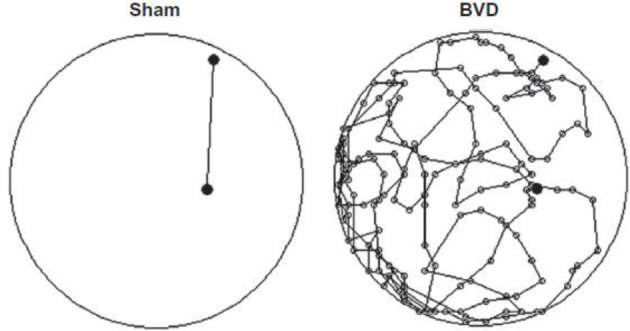
**An example of a homeward path taken by a BVD rat and a sham control rat at 14 months post-op. during a foraging task in darkness in which animals had been trained to forage for food and navigate their way back to a home base**. Whereas the Sham rat traveled the shortest path back home, the BVD rat could not remember the correct path. Reproduced with permission from Baek et al. (2010).

**Figure 7:**
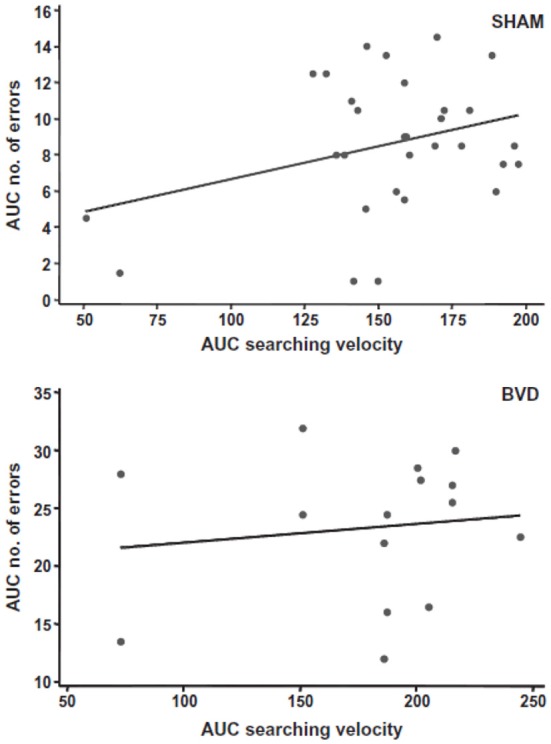
**A scatter graph showing a simple linear regression analysis to predict the number of errors made by all of the sham or BVD animals (in terms of the area under the curve) from their searching velocities in the foraging task, as an index of their locomotor hyperactivity**. There was no significant prediction of spatial memory error from locomotor activity. Reproduced with permission from Baek et al. (2010).

**Figure 8:**
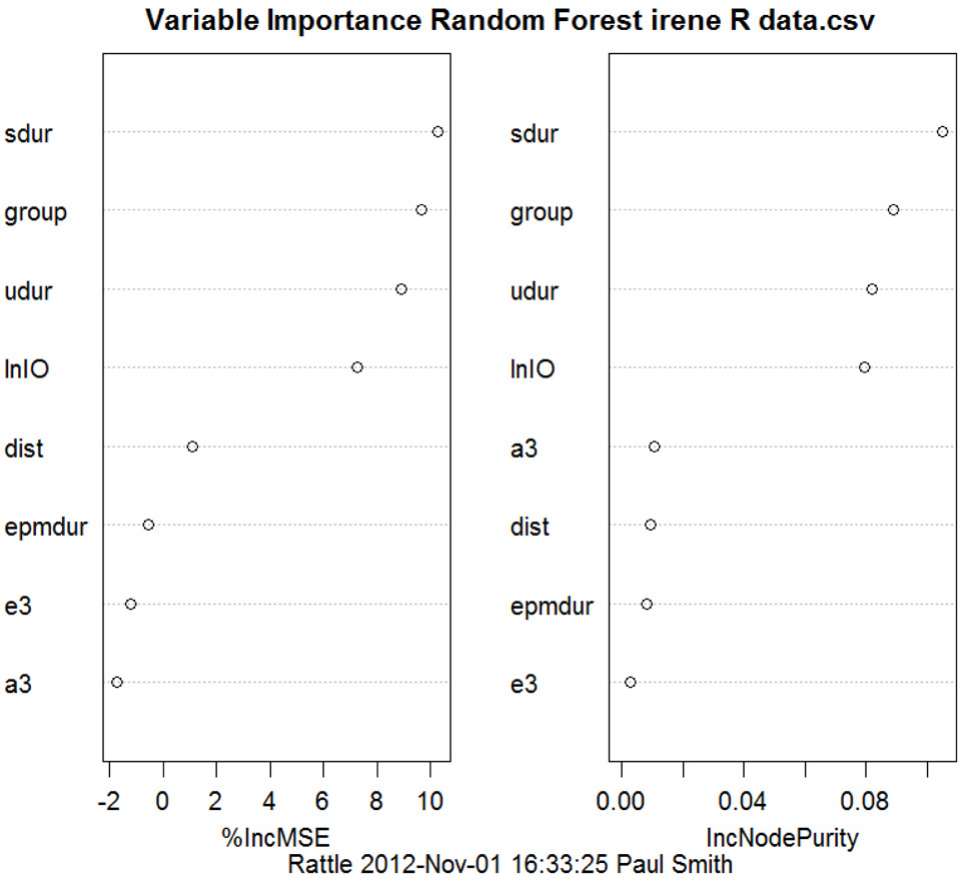
**Random forest regression, showing variables in order of importance from the top to the bottom, to predict the performance of BVD and sham animals in a spatial T maze task from the animals' performance in other behavioral tasks, such as the elevated plus maze, the open field maze and the elevated T maze**. “group:” whether the animals had received a BVD or a sham operation. “epmdur:” duration of open arm entries in the elevated plus maze. “lnIO:” the ln of the ratio of time spent in the inner/middle to the outer zones of the open field maze. “e3:” 3rd escape latency in the elevated T maze. “a3:” 3rd avoidance latency in the elevated T maze. “dist:” distance traveled in the open field maze. “sdur:” duration of supported rearing in the open field maze. “udur:” duration of unsupported rearing in the open field maze. Reproduced with permission from Smith et al. (2013).

Most studies of rats with BVD indicate that they are hyperactive rather than hypoactive (Russell et al., 2003a,b; Goddard et al., 2008; Zheng et al., 2008, 2009a,b, 2012a,b; Baek et al., 2010; Besnard et al., 2012; Stiles et al., 2012; Machado et al., 2012b; see Figure 6). While this makes it difficult to explain poor performance in cognitive tasks in terms of an inability to move, it is conceivable that the hyperactivity prevents the animals from performing accurately in these various cognitive tasks. However, Baek et al. (2010) used regression analyses to show that the poor performance of BVD rats in a foraging task could not be predicted by their hyperactivity (Figure 7). We have recently revisited this issue using multiple linear and random forest regression and found that hyperactivity cannot predict the poor performance of rats in a spatial T maze alternation task, but that the best predictors were whether the animals had received a BVD and the duration of their rearing in an open field maze (Smith et al., 2013; see Figure 8). These results suggest that the poor spatial memory performance of BVD rats cannot be explained by their locomotor hyperactivity. Further evidence in support of this conclusion is that even rats treated with diazepam and exhibiting anxiolytic behavior, still demonstrated the same spatial memory deficits in a radial arm maze task (Machado et al., 2012b).

### Possible relationship between spatial memory deficits and anxiety

**Figure 9:**
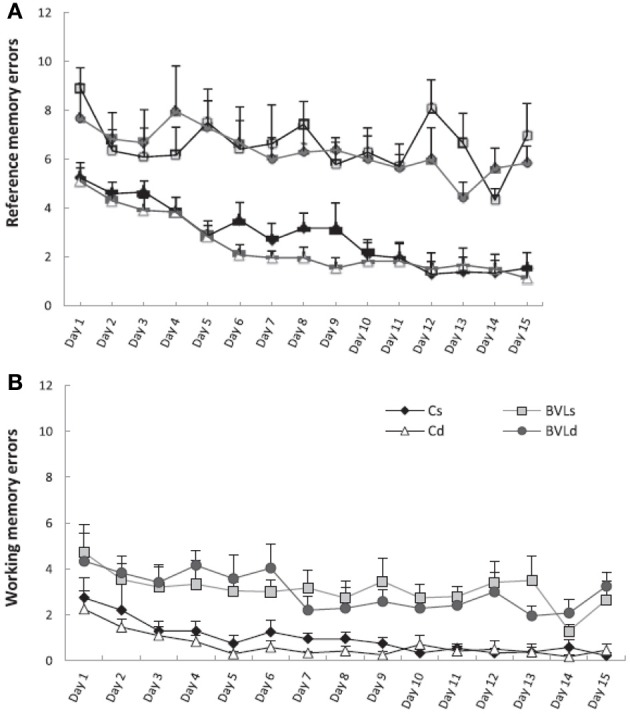
**Effects of 0.5 mg/kg diazepam, which was shown to have an anxiolytic effects, on spatial memory performance in a radial arm maze of bilateral vestibular lesion (BVL) and sham C rats. (A)** Number of reference memory errors. **(B)** Number of working memory errors. Cs, control saline; Cd, control diazepam; BVLs, BVL saline; BVLd, BVL diazepam. Diazepam did not reduce the spatial memory deficits of the BVL rats. Data are expressed as means ± s.e.m. Reproduced with permission from Machado et al. (2012b).

It is possible that the association between vestibular dysfunction and anxiety and depression has some connection with the observed cognitive deficits that accompany vestibular lesions (Balaban and Thayer, 2001; Balaban, 2002; Staab and Ruckenstein, 2003; Staab, 2006). However, at least in animal studies, there is some evidence to disentangle anxiety and cognition in relation to BVD. Machado et al. (2012b) used the black and white box test to show that rats with BVD exhibited increased anxiety and then administered diazepam to reduce it. Although the diazepam appeared to decrease the animals' anxiety, it had no effect on their poor performance in the radial eight-arm maze (Figure 9). Zheng et al. (2012a,b) attempted a similar experiment using the non-benzodiazepine anxiolytic drug, buspirone. In this case, the BVD animals did not exhibit increased anxiety in the elevated plus maze and buspirone had no effect on the time spent in the open arms of the maze, but neither did it have any effect on the animals' poor performance in a spatial forced alternation T maze task. Similarly, an anxiogenic drug, FG-7142, had no effect either on their spatial memory performance. Both of these studies suggest that, at least in animals, spatial memory deficits following BVD may be independent of anxiety.

### Vestibular vs. auditory damage

One of the important limitations of the animal studies in this area is that chemical or surgical lesions of the vestibular labyrinth usually involve damage to the cochlea as well, and therefore it is possible that any cognitive effects of the lesions are partly due to hearing loss. For example, auditory stimulation, including noise trauma, has been reported to affect place cell function (Sakurai, 1990, 1994; Goble et al., 2009). For this reason, in most of our behavioral studies, sham control animals have their tympanic membranes removed so that sound is no longer transmitted effectively to the malleus, incus and stapes. This can serve as only a partial auditory control, however, because some sound will still be transmitted to the cochlea. Nonetheless, we have consistently found that rats without vestibular lesions but with the tympanic membrane removed perform significantly better in cognitive tasks than animals with vestibular lesions (Zheng et al., 2006, 2007, 2008, 2009a,b, 2012b; Baek et al., 2010). This result suggests that hearing loss is not the major cause of the spatial memory deficits in animals subjected to BVD, and is consistent with the results from studies of patients with vestibular dysfunction (e.g., Brandt et al., 2005). In addition, animal studies, using different kinds of aminoglycosides (i.e., streptomycin and neomycin) with different toxicities for the auditory and vestibular hair cells, have shown that the effects of auditory and vestibular lesions on learning and memory are different (Schaeppi et al., 1991). In a radial arm maze task, rats treated with streptomycin, which lesioned the auditory and the vestibular systems, exhibited impaired working memory; however, rats treated with neomycin, which lesioned only the auditory system, did not (Schaeppi et al., 1991). In our tinnitus studies we have found that a unilateral acoustic trauma has no significant effect on spatial memory (Zheng et al., 2011).

It is not clear whether the cognitive deficits associated with vestibular dysfunction in animals are limited to spatial memory impairment. Some animal studies using surgical BVD have also reported deficits in attention (Zheng et al., 2009a) and in object recognition memory that has no spatial component (Zheng et al., 2004). However, others using sequential chemical UVDs have reported no deficits in object recognition memory (Besnard et al., 2012); therefore, this issue remains to be resolved due to methodological differences between the existing studies.

## Human studies of the vestibular lesions on memory

One of the first clinical studies of cognitive function following vestibular loss was reported by Grimm et al. (1989). They described patients with a perilymph fistula syndrome, who reported symptoms such as positional vertigo but also a variety of psychological symptoms, including memory and attention deficits. From a total of 102 patients, more than 85% of them reported memory loss. The patients had a normal level of intellectual function; however, their performance on digit symbol, block design, paired associate learning and picture arrangement tasks, was impaired. Following this, several studies in the 1990's examined the effects of vestibular damage on spatial navigation in humans. They showed that patients with vestibular disorders exhibited deficits in path navigation (Peruch et al., 1999; Cohen, 2000; Cohen and Kimball, 2002; Borel et al., 2004). However, the tasks involved movement, and therefore it could be argued that the deficits resulted from a complex interaction between cognition and postural control. Other studies examined cognitive performance more directly. Black et al. (2004) found that memory problems were common in patients with vestibular loss due to gentamicin ototoxicity. Jauregui-Renaud and colleagues have published a number of studies reporting that patients with vestibular disorders have high rates of depersonalization/derealization symptoms, which include difficulty focussing attention and thoughts seeming blurred (Sang et al., 2006; Jauregui-Renaud et al., 2008a,b; see Gurvich et al., 2013, for review). Other studies have shown that as postural tasks become more challenging, patients with vestibular disorders exhibit deficits in reaction time and memory (Yardley et al., 2001, 2002; Redfern et al., 2004). In a study by Redfern et al. (2004), patients with unilateral vestibular loss showed large increases in complex and inhibitory reaction time, even while seated. A similar result was reported by Talkowski et al. (2005).

### Bilateral vs. unilateral vestibular lesions

**Figure 10:**
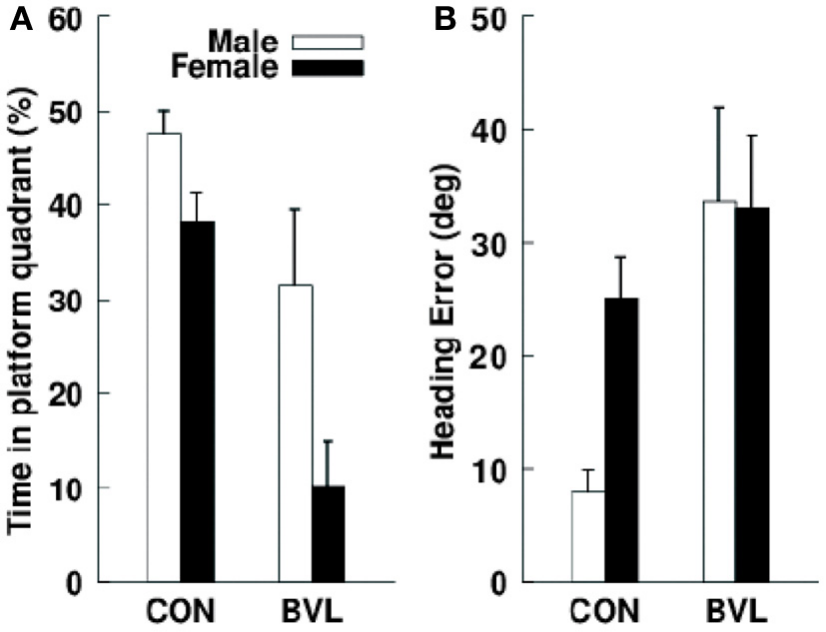
**(A)** Mean percentage search time that male and female control and bilateral vestibular lesion (BVL) patients spent in the correct platform quadrant during the no-platform trial in a virtual Morris water maze task. **(B)** Initial heading error in the same task. Data are expressed as means ± s.e.m. Reproduced with permission from Brandt et al. (2005).

The most sophisticated and well-controlled studies of spatial memory following vestibular lesions in humans, have been reported by Schautzer et al. (2003) and Brandt et al. (2005), who examined the performance in a spatial memory test, the virtual Morris water maze, of 10 patients with bilateral vestibular loss and compared them with age-, sex- and education-matched controls. The task involved moving only a cursor on a computer screen, using a mouse; therefore, performance in the spatial memory task could not have been confounded by VSR deficits. The patients had received bilateral vestibular neurectomies 5–10 years previously for the treatment of neurofibromatosis type 2. They found that the patients with bilateral vestibular loss showed impaired performance in the task when they had to remember a navigation path to a previously visible target; however, they showed no deficit when the target was visible, therefore the effect was specific to memory (see Figure 10). Brandt et al. used the Weschler memory test to show that the patients had normal or above normal non-spatial memory performance. Compared to the controls, the patients also exhibited a significant decrease in the volume of the hippocampus (16.9%). This effect was bilateral, specific to the hippocampus and there was no significant reduction in the total brain volume or in the volume of gray matter or white matter, only a significant increase in cerebrospinal fluid volume. Importantly, only one of the BVD patients had total post-operative hearing loss.

In a study of patients with UVD, Hüfner et al. (2007) reported results from the virtual Morris water maze task that were more complex. During place learning, males with a right UVD and females with a left UVD exhibited a significantly smaller decrease in heading error over the repeated trials compared to the controls and other UVD groups. In addition, inspection of the swim paths showed that only 2 out of the 8 right UVD patients used direct paths to the platform, compared to 12 out of the 16 control subjects, a difference that was statistically significant. In the probe trial, the right UVD patients were found to have a greater heading error than the left UVD patients or control subjects. Finally, in the cued navigation, females with a left UVD performed worse than the other groups in the first visible platform trial. By contrast with the study of Brandt et al. (2005) of patients with BVD, Hüfner et al. (2007) found no significant differences in the hippocampal volume of UVD patients compared to controls. In a later study, Hüfner et al. (2009) reported some subtle deficits in spatial memory in patients with UVD as well as an atrophy of the ipsilateral supramarginal nucleus, the postcentral and superior temporal gyrus and the MT/V5 area, as well as the contralateral thalamus and tegmentum of the mesencephalon. However, zu Eulenburg et al. (2010) reported that patients who had recovered from unilateral vestibular neuritis, exhibited a significant decrease in the volume of the left posterior hippocampus, irrespective of the laterality of the vestibular neuritis. Helmchen et al. (2013) have recently reported the results of a resting state fMRI study of patients with vestibular neuritis. They identified a network of brain regions that was significantly different from controls, including the parietal lobe, the medial aspect of the superior parietal lobule, the posterior cingulate cortex, the middle frontal gyrus, the middle temporal gyrus, the parahippocampal gyrus, the anterior cingulate cortex, the insular cortex, the caudate nucleus, the thalamus and the midbrain.

Consistent with these studies, Hüfner et al. (2010) reported structural changes in the hippocampi of professional dancers and slackliners, who have unusual spatial memory experience. Hufner et al. found that trained subjects exhibited a smaller anterior volume, and a larger posterior volume, in the hippocampal formation, although they showed no difference in spatial memory compared to controls, according to the virtual Morris water maze test.

It is apparent from these studies that the volume of the human hippocampus is sensitive to vestibular input. While the effects of BVD are the most dramatic (Brandt et al., 2005), the effects of unilateral vestibular loss are more circumscribed (Hüfner et al., 2007; zu Eulenburg et al., 2010; Helmchen et al., 2013).

Vestibular disorders have also been reported to impair mental imagery tasks that involve imagined rotations or translations of objects relative to the environment, which are also spatial in nature (Péruch et al., 2011). Grabherr et al. (2011) found that only patients with bilateral vestibular loss, and not unilateral vestibular loss, exhibited impaired ability to mentally transform images of bodies and body parts compared to controls.

It is not entirely clear whether the cognitive deficits associated with vestibular dysfunction in humans are limited to spatial memory impairment. While some studies have reported that other aspects of memory, attention and general intelligence are normal (Schautzer et al., 2003; Brandt et al., 2005), others have reported deficits in attention and concentration (Sang et al., 2006; Jauregui-Renaud et al., 2008a,b) and even dyscalculia (Risey and Briner, 1990-1991; Andersson et al., 2002, 2003; Yardley et al., 2002; see Smith, 2012 for a review).

### Possible connection with vestibular reflex dysfunction and vertigo

As with the animal studies, it is possible that any memory impairment following vestibular damage might simply be a result of vestibular reflex deficits, for example, the inability to see clearly due to oscillopsia or ataxia as a consequence of impaired VORs and VSRs (respectively) (Gizzi et al., 2003). However, studies such as those by Schautzer et al. (2003) and Brandt et al. (2005) were conducted 5–10 years following bilateral vestibular neurectomy. Although the patients would never have recovered normal VOR and VSR function, they would have compensated for the severe acute symptoms (Smith and Curthoys, 1989; Curthoys and Halmagyi, 1995).

Given the severity of some vestibular disorders such as Ménière's disease, it is possible that any cognitive dysfunction is an indirect consequence of symptoms such as vertigo. However, studies of patients with chronic vestibular loss without vertigo, have reported that the patients still exhibit spatial memory impairment (Guidetti et al., 2008). Overall, it seems that patients with at least bilateral vestibular loss, suffer from cognitive problems, particularly spatial memory impairment, that are not simply a direct result of vestibular reflex dysfunction (see Smith et al., 2005a,b; Hanes and McCollum, 2006; Smith et al., 2009, 2010; Gurvich et al., 2013, for reviews).

### Possible connection with anxiety and depression

In the case of humans, it is much more difficult to separate poor performance in cognitive tests from emotional disorders. Vestibular dysfunction in humans is often associated with anxiety disorders, including panic attacks and phobias, as well as depression (Eagger et al., 1992; Asmundson et al., 1998; Balaban and Thayer, 2001; Balaban, 2002; Furman and Jacob, 2001; Monzani et al., 2001; Grunfeld et al., 2003; Persoons et al., 2003; Pollak et al., 2003; Godemann et al., 2004a,b, 2009; Best et al., 2006; Staab, 2006; see Gurvich et al., 2013, for review). Anxiety may be a direct consequence of vestibular dysfunction; however, it has also been reported that anxiety disorders can cause dizziness of vestibular origin (Asmundson et al., 1998; Venault et al., 2001; Bolmont et al., 2002; Staab et al., 2002; Tecer et al., 2004; Best et al., 2006; Furman et al., 2006) and antidepressants such as selective serotonin reuptake inhibitors (SSRIs) have been reported to relieve dizziness associated with anxiety (Staab et al., 2002; Simon et al., 2005; Horii et al., 2007). It possible that emotional disorders arise indirectly from cognitive impairment. However, Halberstadt and Balaban (2006) have reported that the same neurons in the dorsal raphe nucleus that release serotonin, send projections into the amygdala, as well as the brainstem vestibular nucleus. This finding suggests that changes in emotional tone may directly influence the vestibular system.

It is worth noting here that the hippocampus is as much an emotional brain region as one that contributes to spatial memory (Gray and McNaughton, 2003). While the rat dorsal hippocampus (approximately equivalent to the posterior hippocampus in humans) is involved in spatial information processing and spatial memory, the rat ventral hippocampus (approximately equivalent to the anterior hippocampus in humans) processes emotional stimuli (Bannerman et al., 1999, 2004). Therefore, it may be difficult to disentangle the effects of vestibular loss on cognition and emotion.

## Effects of vestibular lesions on head direction cell and place cell function

Taube and colleagues have shown that bilateral inactivation of the vestibular labyrinth, using intratympanic tetrodotoxin, results in the dysfunction of thalamic head direction cells (Stackman and Taube, 1997; see Brown et al., 2002 for a review). In other studies, they have shown that head direction cell activity is degraded during inverted locomotion (Calton and Taube, 2005) and as a result of the loss of vestibular information from either the otoliths (Yoder and Taube, 2009) or the semi-circular canals (Muir et al., 2009). Also using intratympanic tetrodotoxin, Stackman et al. (2002) first reported that loss of vestibular function resulted in a disruption of the selective firing of hippocampal place cells in alert rats. One of the most important aspects of this result was that the disruption to place cell firing patterns was immediate, and it recovered over time, indicating that long-term changes in hippocampal structure were unnecessary for the changes in place cell function. This result was replicated by Russell et al. (2003b) using permanent surgical BVD in rats.

Hippocampal theta rhythm is a large amplitude, quasi-sinusoidal EEG rhythm of ~5–12 Hz which is believed to serve a cohesive function for the firing of hippocampal place cells (Hasselmo, 2005; Vertes, 2005). Many studies in alert animals have reported that theta rhythm in the frequency range of 6–9 Hz can be recorded during movement (see Zou et al., 2009 for a review). In fact, theta frequency has been shown to increase with increasing speed of locomotion (Jeewajee et al., 2008; Lever et al., 2009). Very few studies have investigated the effects of vestibular lesions on theta rhythm. Stackman et al. (2002) investigated theta in one rat in a study in which they transiently inactivated the vestibular system. In the one animal in which they analyzed theta, they found no significant difference from control animals. Russell et al. (2006) used permanent surgical BVD to investigate the effects of vestibular loss on theta rhythm. In contrast to Stackman et al. (2002), they found that hippocampal theta rhythm was severely disrupted. Not only was the power of theta reduced following BVD, but the quasi-sinusoidal character of the waveform was corrupted. Although the BVD animals were hyperactive, theta rhythm was abnormal across the entire range of movement velocities. These results have recently been replicated by Neo et al. (2012), who tried but failed to reverse the spatial memory and emotional deficits caused by BVD by electrically stimulating the septum in order to provide an artificial theta rhythm. Tai et al. (2012) also recently showed that rats that are administered sodium arsanilate intratympanically exhibit a reduction in theta power.

Taken together, these animal studies support the view that vestibular information is important for the generation of spatial memories (Wiener and Berthoz, 1993; Berthoz, 1996; McNaughton et al., 1996, 2006; Etienne and Jeffery, 2004; Smith et al., 2005a,b, 2009, 2010). It is still unclear how vestibular information reaches the hippocampus. Electrical stimulation of one vestibular labyrinth or of the vestibular nucleus has been reported to evoke field potentials, single unit activity and neurotransmitter release in the hippocampus, albeit with a long latency (Horii et al., 1994, 2004; Cuthbert et al., 2000). Caloric or electrical stimulation of the human labyrinth has been shown to cause activation of the hippocampus (Vitte et al., 1996; De Waele et al., 2001) and glucose uptake is reduced in the hippocampus in patients with acute vestibular neuritis (Bense et al., 2001). The thalamus is certain to be one important relay station for the transmission of at least some ascending vestibular information. However, the number of different vestibulo-hippocampal pathways and their precise nature, remains to be determined (Smith, 1997; see Shinder and Taube, 2010 and Hufner et al., 2011 for recent reviews). It must also be kept in mind that the hippocampus is only one part of a highly complex system of limbic-neocortical pathways that are responsible for spatial memory (Guldin and Grusser, 1998; Hanes and McCollum, 2006; Gu et al., 2007; Shinder and Taube, 2010; Lopez and Blanke, 2011). In humans, fMRI has revealed that areas of significant activation by galvanic vestibular stimulation (GVS) include the posterior insula, the retroinsular regions, the superior temporal gyrus, parts of the inferior parietal lobule, the intraparietal sulcus, the post-central and pre-central gyrus, the anterior insular, the inferior frontal gyrus, the anterior cingulate gyrus, the precuneus and the hippocampus (Lobel et al., 1998; see Karnath and Dieterich, 2006 for a review). Activation of cortical networks during GVS is not symmetrical; it appears to be stronger in the non-dominant hemisphere, in the hemisphere ipsilateral to the stimulated ear, and in the hemisphere ipsilateral to the fast phase of vestibular nystagmus (see Karnath and Dieterich, 2006 for a review).

Despite the finding by Brandt et al. (2005) that patients with BVD exhibit a bilateral atrophy of the hippocampus, studies in BVD rats have so far failed to detect such a change. Using a sequential chemical BVD procedure, Besnard et al. (2012) found no significant change in hippocampal volume using MRI. Likewise, Zheng et al. (2012a) could find no change hippocampal volume or neuronal number using stereology. Nonetheless, in unpublished studies, we have found a significant decrease in dendritic length in the hippocampi of BVD rats. One possibility is that hippocampal volume is maintained in rats following BVD due to their locomotor hyperactivity, since movement is known to stimulate hippocampal neurogenesis. Interestingly, Zheng et al. (2012a) observed a significant increase in cell proliferation in the rat hippocampus following BVD. Besnard et al. (2012) also reported an increase in NMDA receptor density and a decrease in affinity in BVD rats. Although Zheng et al. (2013) did not find significant differences in glutamate receptor subunit expression in rats with BVD, principal component analysis did reveal subtle changes in the relationship between different NMDA receptor subunits (Smith and Zheng, 2013). Because Besnard et al. (2012) used autoradioradiography with beta imaging, their results may reflect functional NMDA receptors rather than the total receptor pool.

## Effects of artificial vestibular activation on memory in humans

A small literature exists on the use of low amplitude, noisy GVS to enhance cognition in humans. The overlaying of a noise signal on a galvanic stimulus, termed “noisy GVS,” is based upon the concept of stochastic resonance, in which a sub-threshold sensory stimulus can be made to exceed a fixed threshold if a Gaussian noise signal, with a frequency much higher than the sub-threshold stimulus, is superimposed upon it (Moss et al., 2004).

Bächtold et al. (2001) was the first to report that caloric vestibular stimulation (CVS) could improve verbal and spatial memory in humans, and the effect was greater for right ear irrigation. However, CVS induces vestibular reflexes and therefore it is difficult to separate those effects from cognitive performance. Falconer and Mast (2012) have recently reported that CVS can enhance performance in an egocentric transformation task.

In a later study, Wilkinson et al. (2008) investigated whether noisy, low intensity, bipolar GVS, which was sub-threshold for the activation of the vestibular reflexes, could affect memory for faces. They found that the mean reaction time was shorter for the GVS groups compared to the sham controls, but that the largest reduction was for the anode on the left ear and the cathode on the right ear. They concluded that GVS can improve memory for faces, perhaps by increasing blood flow to the right temporal and parietal cortices. This effect of GVS on memory was unlikely to be due to non-specific arousal since the electrical stimulation was sub-threshold, the memory improvement was greater for the anode on the left, and improvement was found only when a noise signal was added to the GVS. Wilkinson and colleagues have also found that noisy GVS can attenuate prosopagnosia (Wilkinson et al., 2005) and figure copying deficits (Wilkinson et al., 2010). In the most recent study involving two patients with visuo-spatial neglect, GVS was found to have a lasting beneficial effect in a target cancellation task (Zubko et al., 2013). In one of the most systematic studies to date, Dilda et al. (2012) found that suprathreshold GVS significantly increased the error rates for match-to-sample and perspective-taking tasks compared to a subthreshold GVS group; however, reaction time, dual tasking, mental rotation and manual tracking were not significantly affected. Subthreshold GVS had no significant effect on cognitive performance compared to the pre-stimulus conditions.

At the present, there is very little evidence relating to how noisy GVS affects memory. It is not clear whether the effect has any relationship to the effects of vestibular lesions on spatial memory. One possibility is that any beneficial effect of GVS is merely a result of the fact that vestibular information reaches many regions of the neocortex (Bense et al., 2001) and that it will probably cause a change in the integration of sensory information (Wilkinson et al., 2008). However, given the importance of vestibular information to spatial memory, sub-threshold GVS may somehow “prime” the brain to process and store new information. Wilkinson et al. (2012) have recently reported that GVS increased the amplitude of the N170 potential and the power of delta and theta EEG during a face processing task. It is noteworthy that many of these effects of GVS are not restricted to spatial attention or memory. A more recent study by Kim et al. (2013) also supports the idea that GVS modulates neural oscillations.

## Conclusions

Over the last 12 years in particular, a substantial body of evidence has accumulated to suggest that the loss of vestibular function results in cognitive disorders, especially spatial memory deficits that cannot easily be attributed to the direct effects of reflex dysfunction, motor control problems or hearing loss. The spatial memory deficits appear to be most striking in the case of BVD, where there is a complete loss of vestibular function, although there are many more animal studies than human studies to substantiate this. It will be particularly important in future studies to further investigate the effects of unilateral and bilateral vestibular loss in humans, on different types of cognitive function.

In animals at least, the spatial memory deficits appear to be independent of anxiety, although this is less conclusive in humans. One reason to be cautious about disentangling the effects of vestibular damage on cognition and emotion, is that the hippocampus is deeply involved in both (Gray and McNaughton, 2003). While lesions of the dorsal hippocampus in rats result in spatial memory deficits and locomotor hyperactivity, lesions of the ventral hippocampus result in reduced anxiety in the elevated plus maze and hyponeophagia (Bannerman et al., 2002). It is intriguing that BVD, in addition to causing spatial memory deficits and locomotor hyperactivity in rats, has been reported to cause both reduced anxiety in the elevated plus and T mazes (Zheng et al., 2008; Neo et al., 2012) and hyponeophagia (Zheng et al., 2008). This might suggest that BVD results in behavioral effects that are similar to both dorsal and ventral hippocampal lesions and that it may be very difficult to disentangle them completely.

The cognitive effects of vestibular loss appear to be due mainly to the important contribution that the vestibular system makes to neurons involved in spatial navigation and memory, such as head direction cells and place cells, although exactly how this information is used remains to be determined. Information from the otoliths as well as the semi-circular canals seems to be necessary for the normal function of head direction cells; however, so far the different contributions of the otoliths and semi-circular canals to hippocampal place cell function have not been investigated. Furthermore, despite the attention to the thalamus and hippocampus, many different areas of the limbic system and neocortex are involved in these spatial memory processes. Since grid cells in the entorhinal cortex are thought to be responsible for the place fields of hippocampal place cells (see Giocomo et al., 2011 for a review), it might be predicted that BVD would severely disrupt grid cell firing; however, this has not been investigated to date. Future studies will need to specifically address how vestibular information is transmitted to the dorsal and ventral hippocampus in rats and the extent to which otolithic vs. semi-circular canal input is represented in different areas of the hippocampus and entorhinal cortex. At present, we know that vestibular input is necessary for the normal function of the hippocampus but we do not understand how this input is being used.

At present there is some evidence that artificial activation of the vestibular system, using noisy GVS, can modulate memory. However, at present this literature is small and it is unclear whether this effect, when it occurs, is related to the same mechanisms by which vestibular lesions affect memory.

### Conflict of interest statement

The authors declare that the research was conducted in the absence of any commercial or financial relationships that could be construed as a potential conflict of interest.
